# HPV types 16/18 L1 E6 and E7 proteins seropositivity and cervical cancer risk in HIV-positive and HIV-negative black South African women

**DOI:** 10.1186/s13027-022-00418-2

**Published:** 2022-03-29

**Authors:** Mwiza Gideon Singini, Elvira Singh, Debbie Bradshaw, Wenlong Carl Chen, Melitah Motlhale, Abram Bunya Kamiza, Chantal Babb de Villiers, Mazvita Muchengeti, Christopher G. Mathew, Robert Newton, Noemi Bender, Tim Waterboer, Freddy Sitas

**Affiliations:** 1 National Cancer Registry, National Health Laboratory Service, Sandringham, Johannesburg, South Africa; 2 Division of Epidemiology and Biostatistics, School of Public Health, Faculty of Health Sciences, University of the Witwatersrand, Johannesburg, South Africa; 3 Burden of Disease Research Unit, South African Medical Research Council, Cape Town, South Africa; 4 Sydney Brenner Institute for Molecular Bioscience, Faculty of Health Sciences, University of the Witwatersrand, Johannesburg, South Africa; 5 Division of Human Genetics, School of Pathology, Faculty of Health Sciences, University of the Witwatersrand, Johannesburg, South Africa; 6 Department of Medical and Molecular Genetics, Faculty of Life Sciences and Medicine, King’s College London, London, SE1 9RT UK; 7 MRC/UVRI and LSHTM Uganda Research Unit, Entebbe, Uganda; 8 University of York, York, UK; 9 South African DSI-NRF Centre of Excellence in Epidemiological Modelling and Analysis (SACEMA), Stellenbosch University, Stellenbosch, South Africa; 10 Division of Infections and Cancer Epidemiology, German Cancer Research Center (DKFZ), Heidelberg, Germany; 11 Centre for Primary Health Care and Equity, School of Population Health, University of New South Wales, Sydney, Australia; 12 Menzies Centre of Health Policy, School of Public Health, University of Sydney, Sydney, Australia

**Keywords:** Cervical cancer, Human papillomavirus, E6 and E7, L1 proteins, Seropositivity, South Africa

## Abstract

**Background:**

In populations with high rates of human immunodeficiency virus (HIV)-coinfection, the nature of the relationship between human papillomavirus (HPV)-16 and -18 (L1, E6 and E7) antibodies and cervical cancer is still uncertain. We measured the association between seropositivity to HPV (L1, E6 and E7) proteins and cervical cancer among black South African women with and without HIV co-infection.

**Methods:**

We used questionnaire data and serum collected from consecutively recruited patients with a newly diagnosed cancer from the Johannesburg Cancer Study from 1346 cervical cancer cases and 2532 controls (diagnosed with other non-infection related cancers). Seropositivity to HPV proteins was measured using a multiplex serological assay based on recombinant glutathione S-transferase (GST) fusion proteins. We measured associations between their presence and cervical cancer using unconditional logistic regression models and evaluated the sensitivity and specificity of these HPV biomarkers.

**Results:**

Among controls, HIV-negative women from rural areas compared to urban had significantly higher HPV seroprevalence, HPV16 E7 (8.6% vs 3.7%) and HPV18 E7 (7.9% vs 2.0%). HPV16 E6 and E7 antibodies were positively associated with cervical cancer in HIV-positive (Adjusted Odds Ratio (AOR) = 33; 95% CI 10–107) and HIV-negative women (AOR = 97; 95% CI 46–203). In HIV-positive women, HPV E6/E7 antibodies had low sensitivity (43.0%) and high specificity (90.6%) for cervical cancer detection. In HIV-negative women, HPV E6/E7 antibodies sensitivity was 70.6% and specificity was 89.7%.

**Conclusions:**

Our data show that HPV (L1, especially E6 and E7) antibody positivity is associated with cervical cancer in both HIV-positive and HIV-negative women. Nonetheless, being HIV-positive plays an important role in the development of cervical cancer.

**Supplementary Information:**

The online version contains supplementary material available at 10.1186/s13027-022-00418-2.

## Introduction

Globally, in 2018, about 70% of cervical cancers were attributable to high-risk human papillomavirus (HPV) types 16 and 18 [1]. In South Africa, HPV16 and 18 are important causes of cervical cancer and are included in the currently available HPV vaccine.

The HPV early (E6 and E7) oncoproteins and the late (L1) protein are encoded by the HPV genome. The HPV oncoproteins play an important role in the tumorigenesis of cervical cancer [2]. Humoral immune response against major capsid late protein (L1) is generated during infection with HPV; which is a marker of past or present infection [3]. Furthermore, integration of the HPV genome in the host cell results in overexpression of E6 and E7 oncoproteins, which are involved in the transformation and progression of cervical and other HPV-related cancers [4]. Different serological assays have been used to screen for HPV-16 and -18 (E6 and E7) [5–8] oncoprotein antibodies. Serological markers for HPV-antibodies provide useful data about past and current HPV infections and their relationship to cervical cancer [9, 10].

Previous studies have shown that HPV types 16 and 18 L1, E6 and E7 antibody proteins are associated with cervical cancer susceptibility [6, 8, 11–13] however, with varying degrees of strength of association. For example, in a case–control study from Russia, Zumbach et al. [8] utilized an enzyme-linked immunosorbent assay (ELISA) and reported odds ratios (ORs) for cervical cancer of 64.4 (95% CI 3.8–1085) for HPV16 E6 and 4.9 (95% CI 1.3–18.7) for HPV16 E7. In another case–control study from Algeria and India, Combes et al. [10] used a multiplex immunofluorescent HPV serology assay and reported ORs for cervical cancer of 37.1 (95% CI 13.4–103) for HPV16 E6, 12.5 (95% CI 6.3–24.8) for HPV16 E7 and 5.9 (95% CI 3.1–11.1) for HPV16 L1. Earlier work on the Johannesburg Cancer Study (JCS) from 946 human immunodeficiency virus (HIV)-negative women with cervical cancer and 1,342 controls using an ELISA assay and different cut-offs found ORs between moderate and high HPV-16L1 seropositivity of 1.5 (95% CI 1.2–1.9) and 2.4 (95% CI 1.9–3.0) [14]. Thus, multiple serologic methods had been used to characterize the relationship between cervical cancer incidence and HPV.

Less is known about the role of high-risk HPV oncoproteins among cervical cancer patients in black African women who are HIV-positive and HIV-negative. In South Africa, despite high HIV prevalence (24.1%) among black South African women [15], studies on HPV16 and 18 (E6 and E7) and late (L1) seropositivity and the risk of cervical cancer are still lacking. We therefore assessed the seropositivity of HPV -16 and -18 early (E6 and E7), and late (L1) antibodies among cervical cancer cases and infection unrelated cancer controls using a multiplex HPV serology assay [16], and estimated their association with cervical cancer risk among HIV-positive and HIV-negative women.

## Methods

### Setting and participants

The study population were participants recruited into the Johannesburg Cancer Study (JCS) between 1995 and 2016. The aims of JCS included measuring the relative importance of known and emerging risk factors for cancer in a black African population in Johannesburg, South Africa. The details of the JCS have been described elsewhere [17]. Briefly, the JCS collected serum samples from 26,000 consecutive consenting black South African patients with newly diagnosed cancers (> 90% histopathology confirmed), referred to the medical oncology and radiation therapy wards of (the main tertiary public) Charlotte Maxeke Johannesburg Academic Hospital and associated referral hospitals and clinics. Self-reported data on demographics and key lifestyle risk factors were collected using a structured questionnaire. The JCS also collected venous blood in one serum separation tube (SST) and one Ethylenediaminetetraacetic acid (EDTA) tube. SST serum was separated from whole blood within two days of sample collection and the sample was divided into a maximum of 4 aliquots. HIV testing was done using the Vironostika HIV Ag/Ab kit [18]. The study was approved by the University of the Witwatersrand Human Research Ethics Committee (Medical) (certificate number: M200252).

### Cases and controls

**Fig. 1:**
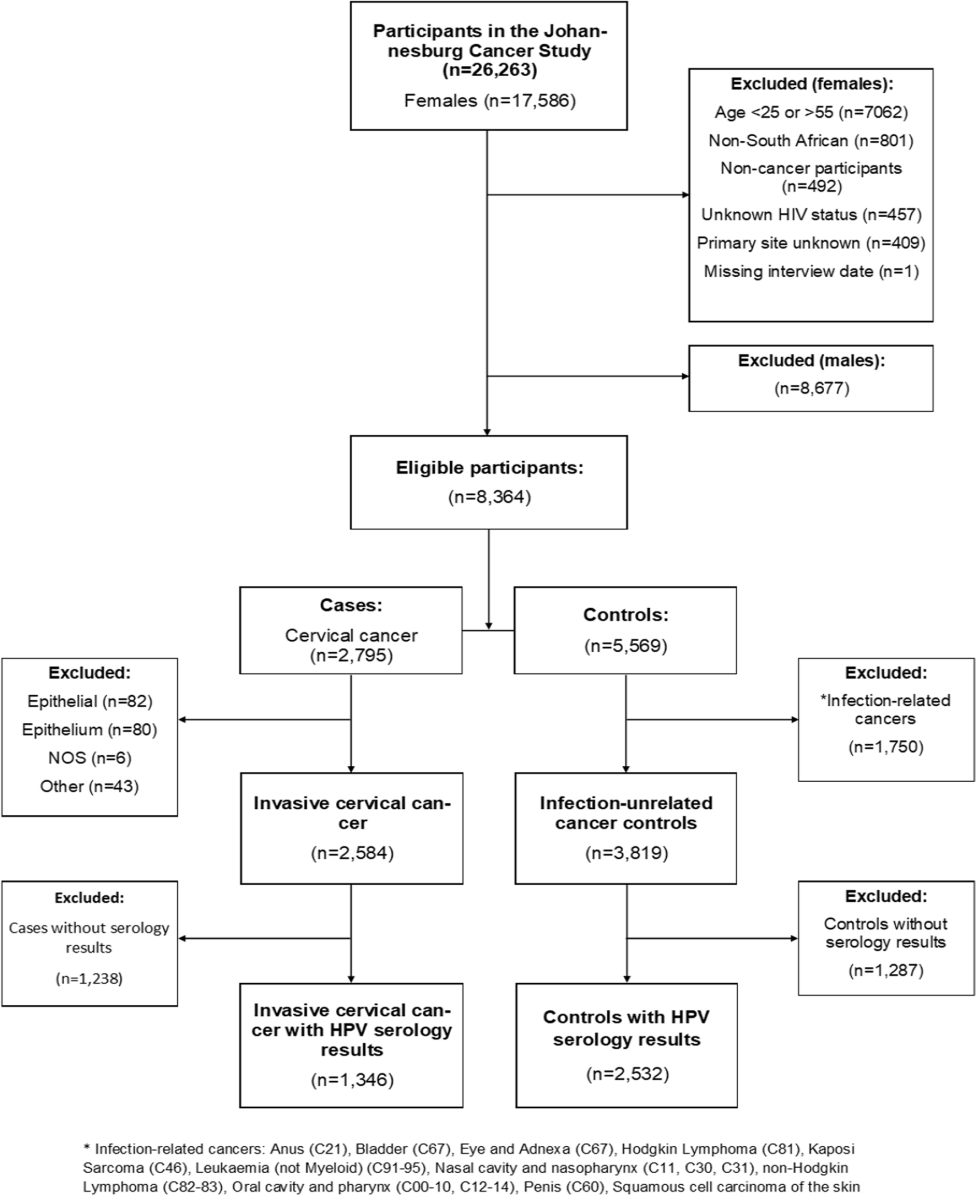
Flow chart of the case–control selection for the subject with HPV-serology results among women aged 25–54 years in the Johannesburg Cancer Study

The JCS is amenable to a case–control design and analysis by selecting controls that are unrelated to the exposures of interest. The current study was restricted to women aged 25 to 54 years because a large proportion of the older women did not have serology samples available. Cases were 1346 women with newly diagnosed cervical cancer (C53). Controls were 2532 women with newly diagnosed infection unrelated cancers (breast (C50) (n = 1953), colon (C18-20) (n = 197), oesophagus (C15) (n = 48), endometrium (C54-55) (n = 75), lung (C33-34) (n = 76), pancreas (C25) (n = 21) and minor cancer types (n = 162) (Additional file 1: Table S1)). Figure 1 outlines the criteria used to select cases and controls.

### Serology data and laboratory methods

Serum aliquots were stored at − 25 °C and one aliquot was shipped on dry ice to the German Cancer Research Center (Deutsches Krebsforschungszentrum (DKFZ)) in Heidelberg, for antibody testing for HPV16 and 18 (L1, E6 and E7) using a multiplex serological assay. This was based on a glutathione S-transferase (GST) capture immunoassay in combination with fluorescent beads on a Luminex platform [16]. The final net (bead and GST background subtracted) Median Fluorescence Intensity (MFI) values of all samples were analysed at a dilution of 1:1000. Previously established standard cut-offs were generated at 1:100 serum dilution [19, 20] and were thus not applicable. Using the Visual Inflection Point (VIP) method [21], we defined net MFI cut-offs of 175 for HPV16 and 18 L1, 75 for HPV16 and 18 E6, 120 for HPV16 E7, and 70 for HPV18 E7. For HPV antibodies, we generated five extra binary variables to describe high-risk combinations for the presence of either E6 /E7 singly or in combination (HPV16 E6 & E7, HPV18 E6 & E7, HPV16&18 E6, HPV16 &18 E7, and HPV16/18 E6/E7).

### Statistical analysis

Data on demographic characteristics were summarized using frequencies and percentages for the categorical variables, medians and the interquartile range (IQR) for the continuous variable that was not normally distributed. A Pearson's Chi-squared test (for categorical variables) and t-test (for age) were used for the comparison of demographics between cases and controls. To assess cross-reactivity between the proteins, a tetrachoric correlation coefficient rank test [22] with Bonferroni adjustments (to take account of multiple comparisons) was used to analyze the correlation between each HPV16 and 18 (L1, E6 and E7) antibodies and HIV antibodies (See Additional file 1: Fig. S2). In the control group, we calculated the seroprevalence of antibodies to HPV16 and 18 (E6 and E7) oncoproteins and L1 protein across different demographic and lifestyle factors, such as age (25–34, 35–44, 45–54 years), place of residence (rural, urban), period of interview (1995–1999, 2000–2004, 2005–2009, 2010–2016), marital status (never married, married, previously married), educational level (none, primary, secondary and above), number of sexual partners (0–1, 2–5, 6+) HIV-status (negative and positive) and parity (0, 1, 2, 3, 4+). We have previously shown that cancer types unrelated to the exposure of interest, resemble background population prevalence for that exposure [18, 23]. We performed a test for heterogeneity on categorical variables and a score test for trends in proportions for ordinal categorical variables. On the assumption that the selected controls should have similar seroprevalences to each other, we compared heterogeneity in seroprevalence of HPV16 and 18 (L1, E6 and E7) antibodies across different control cancer types using the Cochran-Mantel–Haenszel test (See Additional file 1: Fig. S1).

We used an unconditional logistic regression model to assess the association by calculating adjusted Odds Ratios (AOR) and 95% confidence intervals between HPV16 and 18 (E6 and E7) and L1 proteins seropositivity and the risk of cervical cancer. We stratified by HIV status to assess if the seroprevalence of the HPV proteins differs in HIV-positive and HIV- negative patients. We adjusted for age, education level, number of sexual partners, marital status, period of interview and place of residence. To assess whether the clinical performance of HPV-16 and -18 antibodies as a diagnostic marker for cervical cancer we performed the same in HIV-positive and HIV-negative cancer patients. We calculated the sensitivity, specificity of the various HPV antibodies for detecting cervical cancer using the "diagti” command in STATA. In addition, the area under the ROC curve (AUC) of the receiver operator characteristics was computed to compare the performance of the best combination of HPV E6 and E7 antibodies to discriminate cervical cancer from controls (See Additional file 1: Table S4). All the statistical tests were computed using STATA software version 16.0 (Stata Corp, college station, Tx) and SAS v 9.4 (SAS Institute, Cary, NC). All tests were considered significant at a two-tailed alpha of 5%.

## Results

Out of a total of 2,795 cervical cancer cases and 5,569 infection unrelated cancer controls participating in the JCS, 3,878 women (1,346 cases and 2,532 controls) aged 25–54 years were included (Fig. 1). Cases were relatively younger (Median age: 42 (IQR: 37–46) compared to controls (Median age: 44 (IQR: 39–50) (*p* value < 0.001). At least two-thirds of both cases (63.5%) and controls (72.4%) had a secondary school education (Table 1).

**Table 1:** Comparison of demographic characteristics of study participants from the JCS (1995 – 2016)

Characteristics	Total (N = 3878) N (%)	Cervical cancer Cases (N = 1346) n (%)	Controls (N = 2532) n (%)	Comparison of cases and controls *p* value
**Age**				
Median (IQR)	44 (38–48)	42 (37–46)	44 (39–50)	** < 0.001**^**#**^
25–34	528 (13.6)	186 (13.8)	342 (13.5)	** < 0.001**^**$**^
35–44	1,631 (42.1)	693 (51.5)	938 (37.1)	
45–54	1,719 (44.3)	467 (34.7)	1,252 (49.5)	
**Period of interview**				
1995–1999	255 (6.6)	133 (9.9)	122 (4.8)	** < 0.001**
2000–2004	717 (18.5)	254 (18.9)	463 (18.3)	
2005–2009	1,431 (36.9)	538 (40.0)	893 (35.3)	
2010–2016	1,475 (38.0)	421 (31.3)	1,054 (41.6)	
**Number of sexual partners**				
0–1	269 (6.9)	78 (5.8)	191 (7.5)	** < 0.001**
2–5	2,400 (61.9)	929 (69.0)	1,471 (58.1)	
6+	505 (13.0)	192 (14.3)	313 (12.4)	
Unknown	704 (18.2)	147 (10.9)	557 (22.0)	
**Parity**				
0	68 (1.8)	12 (0.9)	56 (2.2)	** < 0.001**
1	632 (16.3)	174 (12.9)	458 (18.1)	
2	1008 (26.0)	335 (24.9)	673 (26.6)	
3	922 (23.8)	325 (24.2)	597 (23.6)	
4+	1,090 (28.1)	473 (35.1)	617 (24.4)	
Missing data	158 (4.1)	17 (2.0)	131 (5.2)	
**Marital Status**				
Never married	1,009 (26.0)	356 (26.5)	653 (25.8)	** < 0.001**
Married	1,941 (50.1)	682 (50.7)	1,259 (49.7)	
Previously married	919 (23.7)	303 (22.5)	616 (24.3)	
Missing data	9 (0.2)	5 (0.4)	4 (0.2)	
**Place of Residence**				
Rural	317 (8.2)	150 (11.1)	167 (6.6)	** < 0.001**
Urban	3,556 (91.7)	1,195 (88.8)	2,361 (93.3)	
Missing data	5 (0.1)	1 (0.1)	4 (0.2)	
**HIV-status**				
Negative	2,535 (65.4)	670 (47.8)	1,865 (73.7)	** < 0.001**
Positive	1,345 (34.6)	676 (50.2)	667 (26.3)	
**Education Level**				
None	278 (7.2)	128 (9.5)	150 (5.9)	**0.016**
Primary	903 (23.3)	358 (26.6)	545 (21.5)	
Secondary and above	2,688 (69.3)	854 (63.5)	1,834 (72.4)	
Missing data	9 (0.2)	6 (0.5)	3 (0.1)	

Controls are cancers unrelated to infection, Ever married includes widowed and divorced, IQR = Interquartile range, ^#^*p* value for a t-test, ^$^*p* value for the chi-squared test

Overall, 50.2% of women with cervical cancer were HIV positive vs. 26.3% HIV positive controls (Table 1). Furthermore, 13.5% of HPV16 L1 seropositive women were also HPV18 L1 seropositive (Additional file 1: Table S3).

**Fig. 2:**
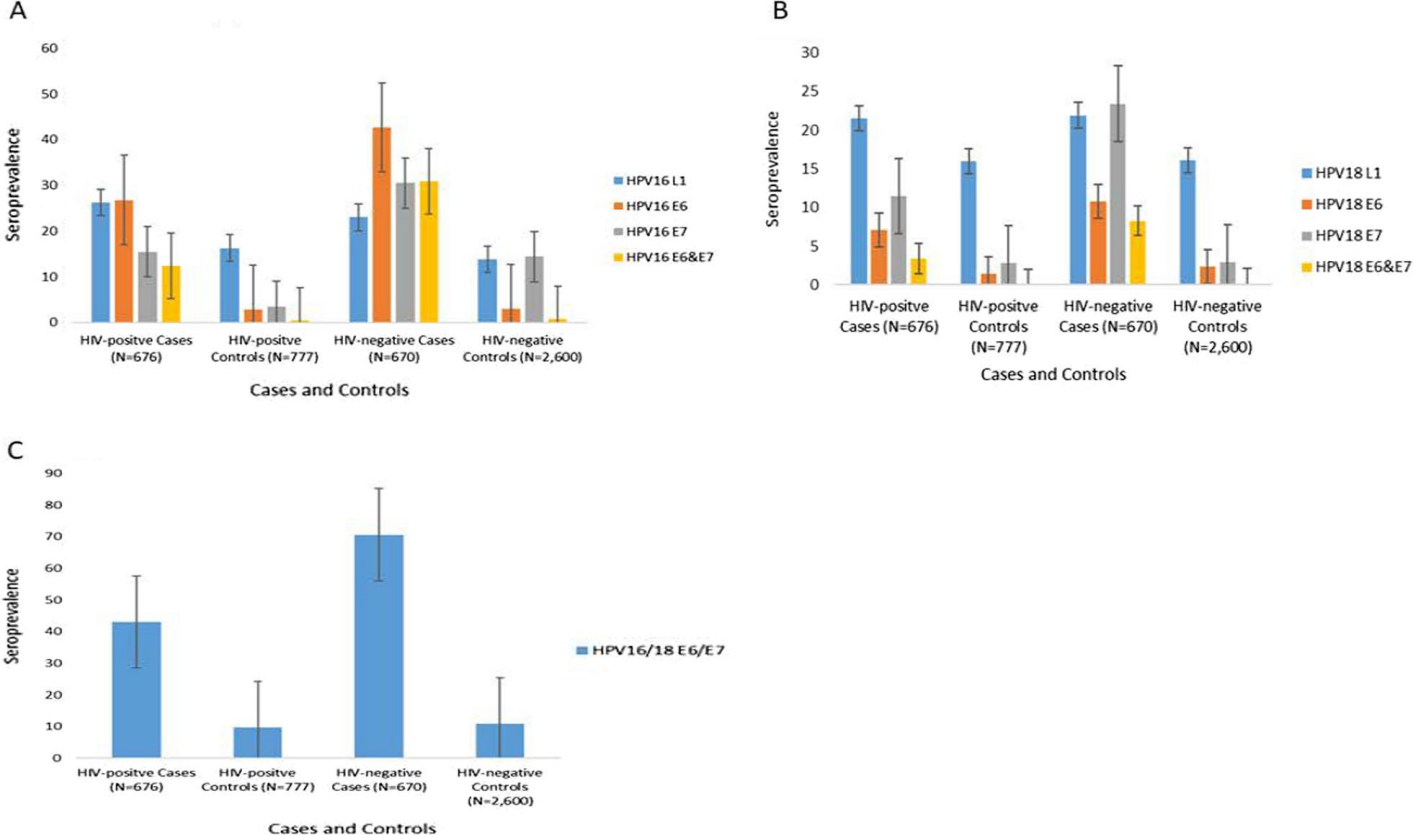
Seroprevalence of HPV-16 and -18 (L1, E6 and E7) proteins antibodies in cervical cancer cases and controls by HIV-status

Stratifying by HIV-status, HPV16 E6 & E7 and HPV18 E6 & E7 seropositivities were higher among HIV-negative women amongst both cases HPV16 E6 (42.7%) and controls HPV16 E6 (3.0%) as compared to HIV-positive women cases HPV16 E6 (26.8%) and controls HPV16 E6 (2.8%) (Fig. 2).

Among HIV-negative controls, the overall antibody seroprevalence for HPV16 L1 was 14.3% and 0.5% for combined HPV16 E6&E7. Similar observations were made for HPV18 L1 (16.3%) and for combined HPV18 E6&E7 (0.2%) (Table 2). In HIV-positive controls, the overall antibody seroprevalence was 15.9% for HPV16 L1, HPV18 L1(15.7%), 0.5% for HPV16 E6 and E7 and 0.2% for HPV 18 E6 and E7 (Table 3).

**Table 2:** Seroprevalence of antibodies to HPV-16 and- 18 L1, E6 and E7 proteins in infection unrelated cancer controls in HIV-negative women

Characteristics		Serology markers							
	Total			HPV-16			HPV-18		
	N = 1865	HPV-16 L1 (%)	HPV-18 L1 (%)	E6 (%)	E7 (%)	E6 & E7 (%)	E6 (%)	E7 (%)	E6 & E7 (%)
**Overall seroprevalence**		14.3	16.3	2.9	4.0	0.5	2.4	2.5	0.2
Demographic									
**Age**									
25–34	206	25 (12.1)	34 (16.5)	2 (1.0)	5(2.4)	1 (0.5)	6 (2.9)	3 (1.5)	0 (0.0)
35–44	638	86 (13.5)	93 (14.6)	19 (3.0)	21 (3.3)	4 (0.7)	17 (2.7)	12 (1.9)	1 (0.2)
45–54	1021	156 (15.3)	177 (17.3)	33 (3.2)	49 (4.8)	3 (0.3)	21 (2.1)	32 (3.1)	3 (0.3)
*Chi-square trend (p value)*		0.166	0.352	0.133	0.053	0.472	0.345	0.067	0.358
**Place of residence**									
Rural	139	18 (13.0)	20 (14.4)	9 (6.5)	12 (8.6)	2 (1.6)	3 (2.2)	11(7.9)	1 (0.8)
Urban	1772	248 (14.4)	284 (16.5)	44 (2.6)	63 (3.7)	6 (0.4)	41 (2.4)	35 (2.0)	3 (0.2)
*Chi-square heterogeneity age-adjusted (p value)*		0.406	0.512	0.202	**0.005**	**0.042**	0.886	** < 0.001**	0.184
**Period of interview**									
1995–1999	115	22 (19.1)	26 (22.6)	5 (4.4)	7 (6.1)	1 (1.0)	4 (3.5)	4 (3.5)	0 (0.0)
2000–2004	373	61 (16.4)	65 (17.4)	15 (4.0)	17 (4.6)	4 (1.2)	5 (1.3)	7 (1.9)	2 (0.6)
2005–2009	663	86 (13.0)	95 (14.3)	19 (2.9)	27 (4.1)	1 (0.2)	15 (2.3)	14 (2.1)	1 (0.2)
2010–2016	714	98 (13.7)	118 (16.5)	15 (2.1)	24 (3.4)	2 (0.3)	20 (2.8)	22 (3.1)	1 (0.16)
*Chi-square heterogeneity age-adjusted (p value)*		0.1757	0.123	0.202	0.455	0.142	0.368	0.538	0.454
**Marital Status**									
Never Married	422	56 (13.3)	67 (15.9)	11 (2.6)	12 (2.4)	2 (0.5)	5 (1.2)	11 (2.6)	0 (0.0)
Married	1002	134 (13.4)	161 (16.1)	30 (3.0)	47 (4.7)	5 (0.5)	34 (3.4)	23 (2.3)	4 (0.4)
Ever married	437	75 (17.2)	75 (17.2)	13 (3.0)	16 (3.7)	1 (0.2)	5 (1.1)	13 (3.0)	0 (0.0)
*Chi-square heterogeneity age-adjusted (p value)*		0.227	0.926	0.976	0.278	0.814	**0.007**	0.798	0.180
**Education Level**									
None	123	22 (17.9)	21(17.1)	6 (4.9)	2 (1.6)	0 (0.0)	4 (3.3)	5 (4.1)	0 (0.0)
Primary	411	54 (13.1)	61 (14.8)	9 (2.2)	20 (4.9)	1 (0.3)	10 (2.4)	8 (2.0)	1 (0.3)
Secondary and above	1329	191 (14.4)	222 (16.7)	38 (2.9)	52 (3.9)	7 (0.6)	30 (2.3)	34 (2.6)	3 (0.2)
*Chi-square adjusted for age trend (p value)*		0.406	0.543	0.298	0.276	0.630	0.654	0.400	0.838
Sexual/ reproductive history									
**Parity**									
0	34	4 (11.8)	5 (14.7)	2 (5.9)	1 (2.9)	1 (3.0)	0 (0.0)	1 (2.9)	0 (0.0)
1	316	30 (9.5)	44 (13.9)	10 (3.2)	15 (4.8)	4 (1.3)	11 (3.5)	9 (2.9)	1 (0.3)
2	485	68 (14.0)	77 (15.9)	18 (3.7)	15 (3.1)	1 (0.2)	13 (2.7)	11(2.3)	0 (0.0)
3	449	81 (18.0)	70 (15.6)	14 (3.1)	18 (4.0)	2 (0.5)	6 (1.3)	11 (2.6)	0 (0.0)
4 +	486	67 (13.8)	88 (18.1)	9 (1.9)	23 (4.7)	0 (0.0)	12 (2.5)	14 (2.9)	3 (0.6)
*Chi-square adjusted for age trend (p value)*		0.143	0.181	**0.031**	0.931	**0.006**	0.610	0.738	0.356
**Number of sexual partners**									
0–1	170	22 (12.9)	25 (14.7)	6 (3.5)	6 (3.5)	0 (0.0)	1 (0.6)	2 (1.2)	0 (0.0)
2–5	1116	161 (14.4)	182 (16.3)	36 (3.2)	53 (4.8)	7 (0.7)	28 (2.5)	31(2.8)	3 (0.3)
6 +	204	32 (15.7)	32 (15.7)	5 (2.6)	6 (2.9)	1 (0.5)	9 (4.4)	5 (2.5)	0 (0.0)
*Chi-square adjusted for age trend (p value)*		0.908	0.522	0.133	0.132	0.339	0.796	0.850	0.862
Unknown	375	52 (13.9)	65 (17.3)	7 (1.9)	10 (2.7)	0 (0.0)	6 (1.6)	9 (2.4)	1 (0.3)

**Table 3:** Seroprevalence of antibodies to HPV-16 and- 18 L1, E6 and E7 proteins in infection unrelated cancer controls in HIV-positive women

Characteristics		Serology markers							
	Total			HPV-16			HPV-18		
	N = 667	HPV-16 L1 (%)	HPV-18 L1 (%)	E6 (%)	E7 (%)	E6 & E7 (%)	E6 (%)	E7 (%)	E6 & E7 (%)
**Overall seroprevalence**		15.9	15.7	2.4	3.3	0.5	1.5	3.2	0.2
Demographic									
**Age**									
25–34	136	24 (17.7)	15 (11.0)	5 (3.7)	5 (3.7)	1 (0.8)	1 (0.7)	1 (0.7)	0 (0.0)
35–44	300	42 (14.0)	50 (16.7)	7 (2.3)	12 (4.0)	1 (0.4)	5 (1.7)	10 (3.3)	0 (0.0)
45–54	231	40 (17.3)	40 (17.3)	4 (1.7)	5 (2.2)	1 (0.5)	4 (1.7)	10 (4.3)	1 (0.5)
*Chi-square trend (p value)*		0.896	0.137	0.254	0.191	0.719	0.490	0.067	0.236
**Place of residence**									
Rural	28	2 (7.1)	4 (14.3)	0 (0.0)	1 (3.6)	0 (0.0)	1 (3.6)	1 (3.6)	0 (0.0)
Urban	639	104 (16.3)	101 (15.8)	16 (2.5)	21 (3.3)	3 (0.5)	9 (1.4)	20 (3.1)	1 (0.2)
*Chi-square heterogeneity age-adjusted (p value)*		0.218	0.797	0.399	0.982	0.724	0.376	0.912	0.856
**Period of interview**									
1995–1999	7	1 (14.3)	1 (14.3)	1(14.3)	0 (0.0)	0 (0.0)	0 (0.0)	0 (0.0)	0 (0.0)
2000–2004	90	9 (10.0)	11 (12.2)	4 (4.4)	4 (4.4)	1 (1.2)	3 (3.3)	1 (1.1)	1 (1.1)
2005–2009	230	32 (13.9)	36 (15.7	5 (2.2)	7 (3.0)	0 (0.0)	4 (1.7)	7 (3.0)	0 (0.0)
2010–2016	340	64 (18.8)	57 (16.8)	6 (1.8)	11 (3.2)	2 (0.6)	3 (0.8)	13 (3.8)	0 (0.0)
*Chi-square heterogeneity age-adjusted (p value)*		0.138	0.872	0.109	0.898	0.572	0.287	0.701	**0.036**
**Marital Status**									
Never Married	231	38 (16.5)	36 (15.6)	4 (1.7)	4 (1.7)	0 (0.0)	1 (0.4)	5 (2.2)	0 (0.0)
Married	257	32 (12.5)	35 (13.6)	8 (3.1)	12 (4.7)	1 (0.4)	4 (1.6)	8 (3.1)	0 (0.0)
Ever married	179	36 (20.1)	34 (19.0)	4 (2.2)	6 (3.4)	2 (1.2)	5 (2.8)	8 (4.5)	1 (0.6)
*Chi-square heterogeneity age-adjusted (p value)*		0.101	0.448	0.573	0.171	0.178	0.188	0.673	0.488
**Education Level**									
None	24	2 (7.4)	1 (3.7)	1 (3.7)	0 (0.0)	0 (0.0)	0 (0.0)	0 (0.0)	0 (0.0)
Primary	134	21 (15.7)	23 (17.2)	2 (1.5)	3 (2.2)	0 (0.0)	4 (3.0)	4 (3.0)	0 (0.0)
Secondary and above	505	83 (16.4)	81 (16.0)	13 (2.6)	19 (3.8)	3 (0.6)	6 (1.2)	17 (3.4)	1 (0.2)
*Chi-square adjusted for age trend (p value)*		0.380	0.169	0.731	0.543	0.611	0.339	0.416	0.701
Sexual/ reproductive history									
**Parity**									
0	22	9 (40.9)	5 (22.7)	0 (0.0)	0 (0.0)	0 (0.0)	0 (0.0)	0 (0.0)	0 (0.0)
1	142	20 (14.1)	15 (10.6)	3 (2.1)	7 (4.9)	0 (0.0)	0 (0.0)	1 (0.7)	0 (0.0)
2	188	38 (20.2)	30 (16.0)	6 (3.2)	8 (4.3)	2 (1.1)	3 (1.6)	8 (4.3)	1 (0.6)
3	148	17 (11.5)	27 (18.2)	3 (2.0)	5 (3.4)	1 (0.7)	4 (2.7)	7 (4.7)	0 (0.0)
4 +	131	17 (13.0)	21 (16.0)	3 (2.3)	2 (1.5)	0 (0.0)	2 (1.5)	4 (3.1)	0 (0.0)
*Chi-square adjusted for age trend (p value)*		0.022	0.688	0.614	0.340	0.932	0.294	0.347	0.373
**Number of sexual partners**									
0–1	21	3 (14.3)	4 (19.1)	1 (4.8)	2 (9.5)	0 (0.0)	0 (0.0)	1 (4.8)	0 (0.0)
2–5	355	55 (15.5)	52 (14.7)	5 (1.4)	9 (2.5)	0 (0.0)	8 (2.3)	8 (2.3)	1 (0.3)
6 +	109	20 (18.4)	19 (17.4)	5 (4.6)	3 (2.8)	1 (1.0)	1 (0.9)	3 (2.8)	0 (0.0)
*Chi-square adjusted for age trend (p value)*		0.869	0.875	0.260	0.518	0.041	0.143	0.291	0.602
Unknown	182	28 (15.4)	30 (16.5)	5 (2.8)	8 (4.4)	2 (1.2)	1 (0.6)	9 (5.0)	0 (0.0)

In general, we did not observe differences in seroprevalence to HPV types 16 and 18 L1 and (E6 and E7) antibodies by demographic and sexual/reproductive factors (*p* value > 0.05). Among HIV-negative controls, the prevalence of HPV16 E7 antibody was higher among women that lived in rural areas (8.6%) as compared to women who lived in urban areas (3.7%) (*p* value for heterogeneity = 0.005) (Table 2). A similar pattern was observed for HPV18 E7 (rural (7.9%) vs urban (2.0%); *p* value = 0.001). Among HIV-positive controls, the prevalence of HPV16 E6 &E7 antibodies decreased with an increase in number births up to 2 births (*p* value = 0.006) (Table 3).

Being seropositive to HPV16 antibodies was significantly associated with cervical cancer: combined HPV16 E6 and 7 (AOR = 69.20, 95% CI 37.07–129.18), HPV16 E6 (AOR = 21.96, 95% CI 16.61–29.02), HPV16 E7 (AOR = 7.93, 95% CI 6.16–10.22) and HPV16 L1 (AOR = 1.74, 95% CI 1.45–2.07). For HPV18, antibody seroprevalence ORs for cervical cancer ranged from AOR = 38.61 (95% CI 15.13–98.53) for combined HPV18 E6&E7, AOR = 8.94 (95% CI 6.64–12.03) for HPV18 E7, AOR = 4.67 (95% CI 3.30–6.50) for HPV18 E6 and AOR = 1.31 (95% CI 1.10–1.56) for HPV18 L1 (Table 4).

**Table 4:** Association between HPV-16 and -18 L1 and (E6 and E7) seropositivity and cervical cancer

Serology markers	Cases (N = 1,346)	Controls (N = 2,532)	Adjusted OR (95% CI)
	n (%) Seropositive	n (%) Seropositive	
HPV16 L1	332 (24.4)	373 (14.7)	1.74 (1.45–2.07)
HPV18 L1	292 (21.7)	409(16.2)	1.31 (1.10–1.56)
**HPV type 16**			
HPV16 E6	467 (35.9)	70 (2.8)	21.96 (16.61–29.02)
HPV16 E7	309 (24.7)	97 (3.8)	7.93 (6.16–10.22)
HPV16 E6 & E7	209 (23.3)	11 (0.5)	69.20 (37.07–129.18)
**HPV type 18**			
HPV18 E6	120 (8.9)	54 (2.1)	4.67 (3.30–6.59)
HPV18 E7	235 (18.6)	68 (2.7)	8.94 (6.64–12.03)
HPV18 E6 &E7	64 (5.9)	5 (0.2)	38.61 (15.13–98.53)
**HPV types 16 or 18**			
HPV16/18 E6/E7	746 (56.8)	255 (10.1)	13.63 (11.32–16.41)

OR adjusted for age, HIV antibodies, education, number of sexual partners, place of residence, marital status and period of interview

**Fig. 3:**
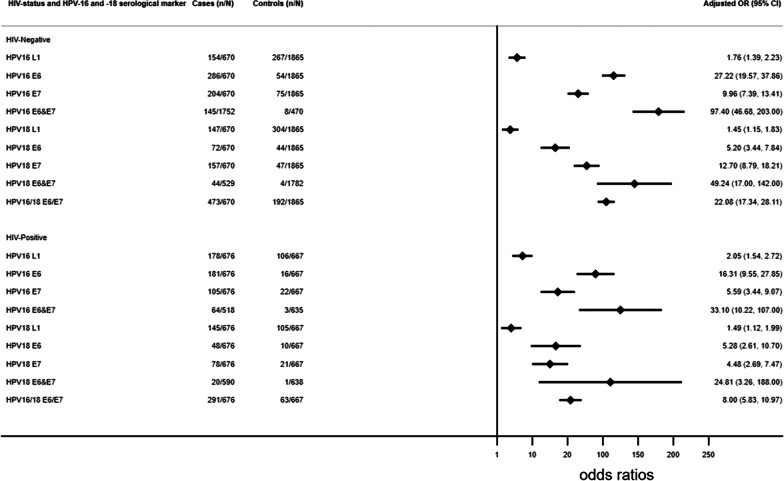
Relationship between HPV-16 and -18 (L1, E6 and E7) seropositivity and cervical cancer risk by HIV-status. Odds ratios adjusted for age, education level, number of sexual partners, marital status place of residence and period of interview

In general, HPV L1 cervical cancer ORs were higher in HIV-positive women but lower in HIV-negative women. The AORs of cervical cancer for combined HPV16 E6 & E7 seropositivity were 97.40 (95% CI 46.68–203.23) in HIV-negative, and 33.10 (95% CI 10.22–107.20) in HIV-positive women (Fig. 3).

Among HIV-positive women, HPV16/18 E6/E7 antibodies had a sensitivity of 43.0%, specificity of 90.6% and AUC of 67% to detect cervical cancer. Among HIV-negative women, the sensitivity, specificity and AUC for HPV16/18 E6/E7 antibodies was 70.6%, 89.7% and 80% respectively for detection of cervical cancer. The sensitivity, specificity and AUC for discriminating cervical cancer based on HPV16 E6 positivity was 26.8%, 97.2% and 62% respectively in HIV-positive women. For HIV-negative women who were HPV E6 seropositive, the sensitivity was 42.7%, specificity was 95.6% and the AUC 69% (Table 5).

**Table 5:** Clinical performance of HPV16 and 18 antibody positivity as diagnostic markers for cervical cancer by HIV-status

Serology markers	HIV-positive			HIV-negative		
	Sensitivity (95% CI)	Specificity (95% CI)	AUC	Sensitivity (95% CI)	Specificity (95% CI)	AUC
HPV16 E6	26.8 (23.5–30.3)	97.6 (96.1–98.6)	62	42.7 (38.9–46.5)	95.6 (94.5–96.4)	69
HPV16 E7	15.5 (12.9–18.5)	96.7 (95.0–97.9)	56	30.4 (27.0–34.1)	96.0 (95.0–96.8)	63
HPV16 E6 & E7	12.4 (9.6–15.5)	99.5 (98.6–99.9)	56	30.9 (26.7–35.2)	99.5(99.1–99.8)	65
HPV18 E6	7.1 (5.3–9.3)	98.5 (97.3–99.3)	53	10.7 (8.5–13.3)	97.6 (96.8–98.3)	54
HPV18 E7	11.5 (9.2–14.2)	97.9 (95.2–98.0)	54	23.4 (20.3–26.8)	97.1 (96.4–97.7)	60
HPV18 E6 & E7	3.4 (2.1–5.2)	99.8 (99.1–100)	52	8.3 (6.1–11.0)	99.9 (99.5–99.9)	54
HPV16/18 E6/E7	43.0 (39.3–46.9)	90.6 (88.1–92.7)	67	70.6 (67.0–74.0)	89.7(88.2–91.0)	80

## Discussion

In this study, we assessed antibody seropositivity to each of HPV-16 and -18 oncoproteins (E6 and E7), and L1 protein and calculated their association with cervical cancer. While the seroprevalence of HPV16 L1 in the previous JCS study among HIV-seronegative women was higher [14] than in this study, ORs between HPV16 L1 and cervical cancer were about the same (overall crude OR 2.2; 95% CI 1.8–2.6, vs 2.1; 1.5–2.7, Fig. 3). Previous data on HPV16 L1 seroprevalence in controls reflect different assay methods and cutoffs used, and perhaps some cross-reactivity with other HPV types. Our key findings, using larger sample size, more recent serological methods measuring a range of high-risk HPV types showed the important role of HPV-16 and -18 (E6 and E7) oncoproteins in cervical cancer risk in both HIV-positive and HIV-negative cancer patients. HPV-16 and -18 (E6 and E7) oncoproteins were associated in cervical cancer risk in both HIV-positive and HIV-negative cancer patients. HIV-positive patients compared to HIV-negative patients had lower sensitivity but with both high specificity for HPV16 and HPV 18(E6 and E7). Findings from our study confirmed that, in HIV-negative controls, HPV16 E7 and HPV18 E7 seroprevalence differed by place of residence. Among HIV-positive women, HPV16 E6&E7 seroprevalence increased with an increase in number of births.

The higher seroprevalence of HPV16 E6, HPV16 E7 and HPV18 E7 in women from rural areas might indicate a greater number of women with early undetected cervical or related cancer lesions. In rural areas of South Africa, women have poorer access to health care services, are less likely to present themselves to cervical cancer screening services and have less access to cancer diagnostic facilities, which are mainly found in tertiary hospitals in urban areas [24].

The high seroprevalence of HPV16 E6 (35.9%) antibodies in cervical cancer cases found in our study are similar to the seroprevalence of HPV16 E6 (32%) in cervical cancer cases reported from the United Kingdom (35%) and India and Algeria (32%) [5, 10]. We found a relatively low seroprevalence of HPV16 E6 & E7 and HPV18 E6 &E7 antibodies among controls in keeping with a low prevalence of HPV oncoprotein antibodies among women without cervical cancer [25]. Our study demonstrates that the high seroprevalence of HPV16 E6 and E7 antibodies in cervical cancer development is in accordance with other studies from Russia and Italy [8, 26].

Antibodies to the E6 and E7 oncoproteins are late markers of invasive cervical cancer [27]. In our study, seropositivity to each of the HPV-16 and -18 (E6 and E7) antibodies was associated with high risks of cervical cancer. Notably, seropositivity to HPV16 (E6 & E7) and HPV18 (E6 & E7) exhibited the highest ORs for cervical cancer (Fig. 3). Similar findings were reported in a case–control study of patients recruited from India and Algeria [10]. Our findings support those from other cross-sectional, case–control and prospective studies, albeit each using different assays [27–30].

Individuals with suppressed immune systems are susceptible to chronic infection, including HPV [31]. Thus, HIV infection increases the risk of HPV persistence [32]. Existing evidence of the association between HPV (E6 and E7) and L1 proteins antibodies and HIV has come from studies on men who have sex with men [10, 33, 34]. In contrast, most of the existing studies on the association between antibodies to HPV-16 and -18 L1, E6 and E7 proteins and cervical cancer risk have been conducted in low HIV prevalence settings [6, 8, 13, 27]. In our study, access to HIV data made it possible to stratify our analysis by HIV status. Our finding showed that antibody seropositivity to each of HPV-16 and -18 (E6 and E7) oncoproteins was strongly associated with the risk of cervical cancer among HIV-positive women. Our data support the hypothesis that HIV plays an important role in the persistence of HPV infection.

Unexpected findings in our study were that HPV-16 and -18 (E6 and E7) oncoproteins seropositivity and cervical cancer risk were higher in HIV-negative cancer patients compared to HIV-positive cancer patients. Similar findings have been observed in previous studies of HPV antibodies among HIV-positive and HIV-negative patients [35–38]. In our study, the possible explanation could be that cervical cancer risk in HIV positive women may be related to more than the two main types HPV16 and HPV18 that we tested for so their relative contribution appears attenuated (Additional file 1: Table S3). Other studies on HPV genotype distribution by HIV-status that were conducted in South Africa reported that HPV types 35, 58 and 33 were more commonly identified [39, 40] in addition to types 16 and 18. However, if this is indeed true, then the current HPV vaccine (16/18) may be less effective for HIV positive women. Another possible explanation is that in HIV-positive patients, antibodies could be an indication of reactivation of a latent HPV while in HIV-negative patients antibodies could be reinfection with a new type [35]. Another unexpected finding in our study was that the number of sexual partners was not associated with HPV L1 and (E6 and E7) antibodies. The plausible explanation could be that peak acquisition of HPV occurs at an age earlier than the age of participants in our study [41].

In the new guidelines for screening cervical cancer, the World Health Organization (WHO) has included HPV antibodies and oncoproteins as a future potential screening test [42]. We, therefore, assessed the clinical performance of HPV E6 /E7 antibodies as possible screening tests. We found that the sensitivity of the HPV antibodies for detection of cervical cancer was low but the specificity was high among HIV-positive women. In contrast, sensitivity and specificity for the detection of cervical cancer were high among HIV-negative women. Predictive values would thus vary considerably depending on the background prior likelihood of disease. Our finding is in line with the previous findings on the sensitivity and specificity of HPV16/18 E6/E7 antibodies as a screening test [28, 43].

There are strengths to our study. The seroprevalence of HPV antibodies was conducted on a large sample size, and we used a high throughput multiplex serology so all tests were done under the same laboratory conditions. The laboratory tests were conducted ‘blind’ without prior knowledge of the case/control status of the participants. Nonetheless, our study has some limitations. Serology data on exposure and cervical cancer diagnoses (of all stages) were collected at the same time. We did not have tumor HPV Deoxyribonucleic Acid (DNA) data to determine the causative type(s). Since only 50–70% of women with detectable HPV DNA in the cervix seroconvert [44], our study underestimates the true prevalence of HPV infection. The JCS did not recruit women with cervical pre-cancerous lesions. Study results are based on black women aged 25- 54 years who were recruited from the catchment area of the largest public tertiary hospital in Johannesburg. Therefore, our findings might not be generalizable to other South African regions and women aged 55 years and above.

## Conclusions

Our data contribute to the evidence on the importance of HPV-16 and -18 E6 and E7 oncoproteins antibodies in discriminating cervical cancer from controls in a black South African population. HPV L1 antibodies show to be exposure markers of past HPV infection. HPV E6 and E7 show to be important markers of invasive cervical cancer. Furthermore, antibodies to HPV-16 and-18 E6 and E7 seropositivity are strongly associated with cervical cancer in both HIV-positive and HIV-negative patients.

## Supplementary Information

**Additional file 1: Table S1:** Comparison of HPV16 and 18 (E6 and E7, L1) antibodies in cervical cancer Cases and Controls among young and older women. **Table S2:** Seroprevalence of HPV16 and 18 L1. **Table S3:** Seroprevalence of antibodies against HPV 16 and 18 (L1, E6 and E7) by HIV-Status. **Table S4:** Clinical performance of HPV16 and 18 antibodies as a diagnostic marker for cervical cancer. **Fig. S1:** Age-adjusted seroprevalence of HPV related antibody markers in cervical cancer cases and other infection unrelated cancer controls and p-value for heterogeneity among the infection unrelated cancer controls (i.e. breast, colon, oesophagus, endometrium lung, pancreas, other minor types). The seropositivity of the age-adjusted HPV L1 and (E6 and E7) antibodies in each of the infection unrelated cancer patients (controls) was similar, (p-heterogeneity > 0.05). **Fig. S2:** Correlation between HPV16 and 18 proteins L1, E6 and E7 antibodies and HIV antibodies, R-values > 0.8 shows high correlation. (* Significance at 0.05). The colour indicates the intensity of correlation. Green indicate positive values, red indicate negative values, orange shows R-values that are greater than 0.05 and dark orange indicate R-values < 0.05 but not statistically significant.

## Data Availability

Data cannot be shared publicly because of ethics policy at University of Witwatersrand, whereby any new analyses require Human Research Ethics Committee approval. Data are available from the SA-NCR /National Health Laboratory Services. (contact: adrianp@nicd.ac.za) for researchers who meet the relevant ethics criteria for access to these data.

## Abbreviations

AOR: Adjusted odds ratio
AUC: Area under the curve
CI: Confidence interval
DFKZ: Deutsches Krebsforchungszentrum
DNA: Deoxyribonucleic acid
ELISA: Enzyme-linked immunosorbent Assay
EDTA: Ethylenediaminetetraacetic acid
GST: Glutathione S-transferase
HIV: Human immunodeficiency virus
HPV: Human papillomavirus
IQR: Interquartile range
JCS: Johannesburg Cancer Study
SST: Serum separation tube
MFI: Median fluorescence intensity
ROC: Receiver operating characteristic
USA: United States of America
VIP: Visual inflection point
WHO: World Health Organization
