# Supplementary material for: HPV types 16/18 L1 E6 and E7 proteins seropositivity and cervical cancer risk in HIV-positive and HIV-negative black South African women
Source: Infect Agent Cancer. 2022 Mar 29;17:14. doi: 10.1186/s13027-022-00418-2 (PMC8966297; doi:10.1186/s13027-022-00418-2)
Supplement: Supplementary file 1 — Additional file 1: Table S1: Comparison of HPV16 and 18 (E6 and E7, L1) antibodies in cervical cancer Cases and Controls among young and older women. Table S2: Seroprevalence of HPV16 and 18 L1. Table S3: Seroprevalence of antibodies against HPV 16 and 18 (L1, E6 and E7) by HIV-Status. Table S4: Clinical performance of HPV16 and 18 antibodies as a diagnostic marker for cervical cancer. Fig. S1: Age-adjusted seroprevalence of HPV related antibody markers in cervical cancer cases and other infection unrelated cancer controls and p-value for heterogeneity among the infection unrelated cancer controls (i.e. breast, colon, oesophagus, endometrium lung, pancreas, other minor types). The seropositivity of the age-adjusted HPV L1 and (E6 and E7) antibodies in each of the infection unrelated cancer patients (controls) was similar, (p-heterogeneity > 0.05). Fig. S2: Correlation between HPV16 and 18 proteins L1, E6 and E7 antibodies and HIV antibodies, R-values > 0.8 shows high correlation. (* Significance at 0.05). The colour indicates the intensity of correlation. Green indicate positive values, red indicate negative values, orange shows R-values that are greater than 0.05 and dark orange indicate R-values < 0.05 but not statistically significant. [file 13027_2022_418_MOESM1_ESM.docx]

**Additional files: Tables and Figures**

Additional file1. Supplementary Table 1: Comparison of HPV16 and 18 (E6 and E7, L1) antibodies in cervical cancer Cases and Controls among young and older women

|  | Young adult women (Age 25-34 years) | | | Older adult women (Age 35-54) | | |
| --- | --- | --- | --- | --- | --- | --- |
|  | Cases (N=186) | Controls (N=342) |  | Cases (N=1160) | Controls (N=2,190) |  |
| Serology markers | n (%)  seropositive | n (%)  seropositive | Odds Ratios (95%CI) | n (%)  seropositive | n (%)  seropositive | Odds Ratios (95%CI) |
| HPV16 L1 | 52 (28.0) | 49 (14.3) | 1.89 (1.17-3.04) | 280 (24.1) | 324 (14.8) | 1.66 (1.37-2.01) |
| HPV16 E6 | 62 (33.3) | 7 (2.1) | 28.15 (11.77-67.32) | 405 (34.9) | 63 (2.9) | 20.90 (15.60-28.00) |
| HPV16 E7 | 36 (19.4) | 10 (2.9) | 9.18 (4.13-20.42) | 273 (23.5) | 87 (4.0) | 7.39 (5.68-9.62) |
| HPV16 E6&E7 | 22 (16.7) | 2 (0.6) | 40.13 (8.59-187.48) | 187 (21.9) | 9 (0.4) | 72.41 (36.57-143) |
| HPV18 L1 | 45 (24.2) | 49 (14.3) | 1.51 (0.92-2.47) | 247 (21.3) | 360 (16.4) | 1.24 (1.03-1.51) |
| HPV18 E6 | 16 (8.6) | 7 (2.1) | 4.71 (1.80-12.34) | 104 (9.0) | 47 (2.2) | 4.69 (3.25-6.77) |
| HPV18 E7 | 25 (13.4) | 4 (1.2) | 17.15 (5.43-54.17) | 210 (18.1) | 64 (2.9) | 8.36 (6.16-11.34) |
| HPV18 E6&E7 | 8 (5.0) | 0 (0.0) | N/A | 56 (5.9) | 5 (0.2) | 33.70 (13.17-86) |
| HPV16&18 E6 | 13 (9.7) | 0 (0.0) | N/A | 36 (5.0) | 5 (0.2) | 25.61 (9.78-67) |
| HPV16 &18 E7 | 2 (1.6) | 0 (0.0) | N/A | 32 (4.3) | 8 (0.4) | 11.59 (5.14-26.13) |
| HPV16/18 E6/E7 | 93 (50.0) | 26 (7.6) | 13.11 (7.59-22.67) | 67 1(57.8) | 229(10.5) | 13.34 (10.98-16.21) |
| Odds ratios adjusted for HIV-antibodies, education level, number of sexual partners,marital status, place of residence and period of interview | | | | | | |

Additional file 2. Supplementary Table 2: Seroprevalence of HPV16 and 18 L1

| HPV L1 (16&18) | Total (Col %) |
| --- | --- |
| Negative | 2,929 (86.5) |
| Positive | 457 (13.5) |

Additional file 3. Supplementary Table 3: Seroprevalence of antibodies against HPV 16 and 18 (L1 E6 and E7) by HIV-Status

| seropositive | HIV-positive  n (%) | HIV-negative  n (%) |
| --- | --- | --- |
| HPV L1 (16&18) | 169 (14.7%) | 288 (12.9) |
| HPV E6 (16&18) | 24 (2.1) | 30 (1.4) |
| HPV E7 (16&18) | 9 (0.8) | 33 (1.6) |

Additional file 4. Supplementary Table 4: Clinical performance of HPV16 and 18 antibodies as a diagnostic marker for cervical cancer

|  | Diagnostic | | |
| --- | --- | --- | --- |
| Serology markers | Sensitivity  (95% CI) | Specificity  (95% CI) | AUC |
| HPV16 E6 | 34.7 (32.2-37.3) | 97.0 (96.4-97.6) | 66 |
| HPV16 E7 | 23.0 (20.7-25.3) | 96.2 (95.3-96.9) | 60 |
| HPV16 E6&E7 | 21.2 (18.6-23.8) | 99.5 (99.2-99.8) | 60 |
| HPV18 E6 | 8.9 (7.4-10.6) | 97.8 (97.3-98.3) | 53 |
| HPV18 E7 | 17.5 (15.5-19.6) | 97.3 (96.6-97.9) | 57 |
| HPV18 E6&E7 | 5.7 (4.4-7.2) | 99.8 (99.5-99.9) | 53 |
| HPV16/18 E6/E7 | 56.8 (54.1-59.4) | 89.3 (88.2-90.4) | 73 |


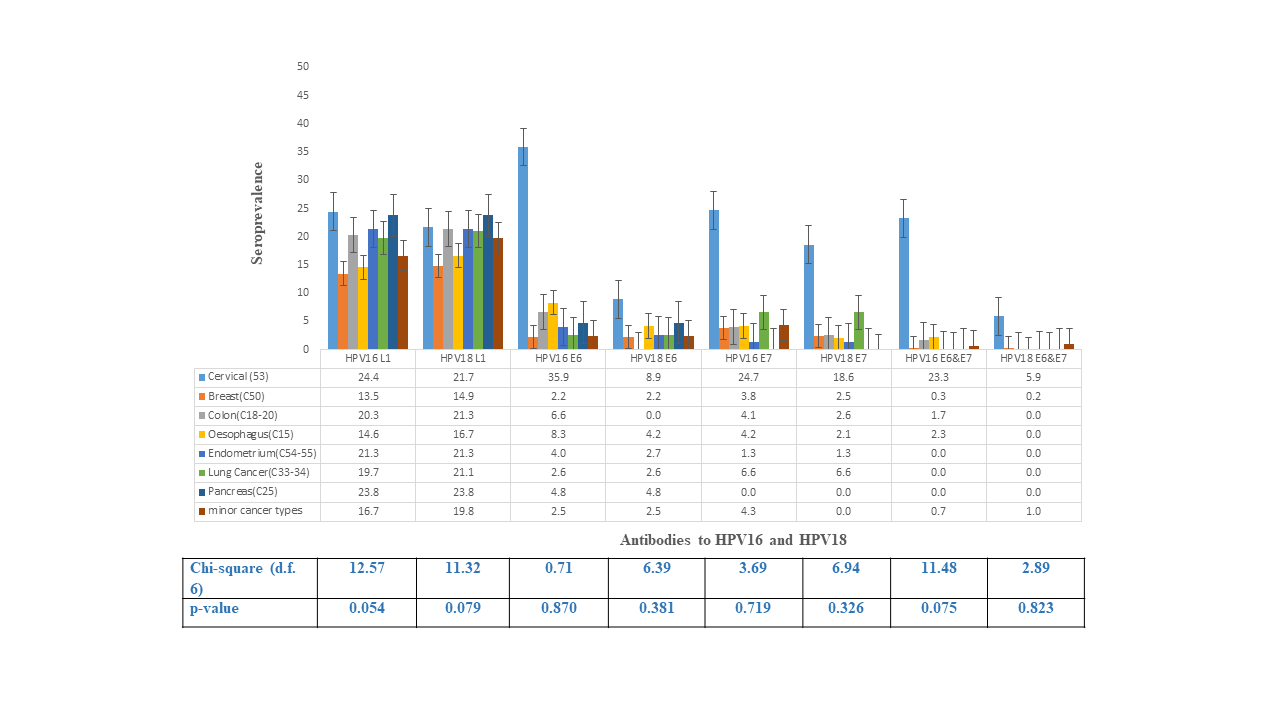


Additional file 5. Supplementary Fig. 1: Age-adjusted seroprevalence of HPV related antibody markers in cervical cancer cases and other infection unrelated cancer controls and p-value for heterogeneity among the infection unrelated cancer controls (i.e. breast, colon, oesophagus, endometrium, lung, pancreas, other minor types). The seropositivity of the age-adjusted HPV L1 and (E6 and E7) antibodies in each of the infection unrelated cancer patients (controls) was similar, (p-heterogeneity >0.05).

Additional file 6. Supplementary Fig. 2: Correlation between HPV16 and 18 proteins L1, E6 and E7 antibodies and HIV antibodies, R-values > 0.8 shows high correlation. (* Significance at 0.05). The colour indicates the intensity of correlation. Green indicate positive values, red indicate negative values, orange shows R-values that are greater than 0.05 and dark orange indicate R-values < 0.05 but not statistically significant.
